# Connexin 32 and connexin 43 are involved in lineage restriction of hepatic progenitor cells to hepatocytes

**DOI:** 10.1186/s13287-017-0703-2

**Published:** 2017-11-07

**Authors:** Haiyun Pei, Chao Zhai, Huilin Li, Fang Yan, Jinhua Qin, Hongfeng Yuan, Rui Zhang, Shuyong Wang, Wencheng Zhang, Mingyang Chang, Yunfang Wang, Xuetao Pei

**Affiliations:** 1 Stem Cell and Regenerative Medicine Lab, Beijing Institute of Transfusion Medicine, Beijing, 100850 China; 2 Tissue Engineering Lab, Beijing Institute of Transfusion Medicine, Beijing, 100850 China; 3 South China Institute of Biomedicine, Guangzhou, 510005 China

**Keywords:** Connexin, p38 MAPK, Hepatic progenitor cell, Hepatocyte, Differentiation, Cell therapy

## Abstract

**Background:**

Bi-potential hepatic progenitor cells can give rise to both hepatocytes and cholangiocytes, which is the last phase and critical juncture in terms of sequentially hepatic lineage restriction from any kind of stem cells. If their differentiation can be controlled, it might access to functional hepatocytes to develop pharmaceutical and biotechnology industries as well as cell therapies for end-stage liver diseases.

**Methods:**

In this study, we investigated the influence of Cx32 and Cx43 on hepatocyte differentiation of WB-F344 cells by in vitro gain and loss of function analyses. An inhibitor of Cx32 was also used to make further clarification. To reveal p38 MAPK pathway is closely related to Cxs, rats with 70% partial hepatectomy were injected intraperitoneally with a p38 inhibitor, SB203580. Besides, the effects of p38 MAPK pathway on differentiation of hepatoblasts isolated from fetal rat livers were evaluated by addition of SB203580 in culture medium.

**Results:**

In vitro gain and loss of function analyses showed overexpression of Connexin 32 and knockdown of Connexin 43 promoted hepatocytes differentiation from hepatic progenitor cells. In addition, in vitro and ex vivo research revealed inhibition of p38 mitogen-activated protein kinase pathway can improve hepatocytes differentiation correlating with upregulation of Connexin 32 expression and downregulation of Connexin 43 expression.

**Conclusions:**

Here we demonstrate that Connexins play crucial roles in facilitating differentiation of hepatic progenitors. Our work further implicates that regulators of Connexins and their related pathways might provide new insights to improve lineage restriction of stem cells to mature hepatocytes.

**Electronic supplementary material:**

The online version of this article (doi:10.1186/s13287-017-0703-2) contains supplementary material, which is available to authorized users.

## Background

With multiple functions of synthesis and detoxification, hepatocytes are great value to promote development of the pharmaceutical and biotechnology industries as well as cell transplantation for the treatment of end-stage liver diseases [1]. However, its application has been terribly impeded by limitations and scarcity of donor tissue, inability of proliferation, difficulties in cryopreservation, deprivation of functions and de-differentiation in vitro [2]. An alternative that is becoming available is to generate fully functional hepatocytes from lineage restriction of stem cells. With pluripotency and unlimited self-renewal capacity, pluripotent stem cells (PSCs), including embryonic stem cells (ESCs) and induced pluripotent stem cells (iPSCs), could be ideal sources of hepatocytes for clinical and industrial applications [3–6]. Nevertheless, so far, PSCs-derived hepatocytes usually possessed fetal hepatocyte-like phenotype and functions, which indicated that the key step for the final stage from hepatic progenitor cells to functional maturation of hepatocytes was the major challenge among the step-wised differentiation [7]. Consequently, it is highly necessary to make further clarification on mechanism of hepatic progenitors’ differentiation to develop optimized strategy for derivation of hepatocytes.

Gap junctions are the pores coupling adjacent cells to mediate intercellular activities of gap junctional intercellular communication (GJIC), which are thought to function as a channel for exchange of materials between cells [8]. They are formed by connexons, “iris-diaphragm-like” structures composed of six connexin (Cx) protein subunits. The Cx family is divided into 13 different members according to molecular weight. Previous research uncovered crucial roles of Cxs in stem cell proliferation and differentiation, for instance, modulation of Cx43 involved in differentiation of osteoblast [9], odontoblast [10], and lens epithelial cells [11].

In adult liver, Cx32, occupied approximately 90% of Cxs on hepatic parenchymal cells, establishes an elaborate GJIC network between hepatocytes and becomes indispensable for liver development [12]. Dozens of studies have confirmed that Cx32 expression was strongly and positively correlated with multiple functions of hepatocytes, including glycogenolysis [13, 14], albumin (Alb) secretion [15], ammonia detoxification [15], bile secretion [16], and CYP (cytochrome P450)-mediated biotransformation [17–19]. What is more notable is that Cx expression patterns undergo lineage stage-dependent transformation in embryonic liver. Especially, a switch from Cx43 to Cx32 expression has been proved upon differentiation from hepatic progenitor cells to hepatocytes [12, 20–22], which dropped us a hint that Cxs might signal commitment to hepatocytes differentiation from early progenitor cells in the liver. Besides, they may interact with other proteins as upregulators or down-effectors to play significant roles.

The p38 mitogen-activated protein kinase (p38 MAPK) pathway has been reported to be involved in the regulation of many stem cell types. In particular, it is essential for the proper differentiation of stem cells in hematopoietic [23], muscular [24], neural [25, 26], cardiac [27], pancreatic [28–30], lung [31], and skin [32, 33] tissues. Former studies showed that inhibition of p38 MAPK activity could regulate Cx32 or Cx43 in both rat neuronal stem cells and liver epithelial cells [34–36]. Moreover, in vivo research indicated that downregulation of Cx32 protein after partial hepatectomy could be reversed by the treatment of p38 MAPK inhibitor [37]. Last year, our lab further revealed that Cx32-mediated GJIC was necessary for hepatocyte differentiation of human ESCs during step-wise hepatic lineage restriction and p38 MAPK inhibitor improved maturation of differentiated cells correlating with upregulation of Cx32 through inhibition of phosphorylation-dependent degradation of Cxs [38].

Although the shift phenomenon of Cxs in liver development is well known, the function of Cxs in differentiation of hepatic progenitor cells and the relationship with p38 MAPK pathway remains largely unknown. In this study, we explored the function of Cxs by using WB-F344 cells (WB cells), a rat cell line with hepatic progenitor-like features, which has often been used as an in vitro model to study the differentiation of hepatic progenitor cells into hepatocytes [39–42]. In vitro gain and loss of function analyses showed overexpression of Cx32 and knockdown of Cx43 promoted hepatocyte differentiation from hepatic progenitor cells. Furthermore, p38 inhibitor, SB203580, can improve hepatocyte differentiation correlating with upregulation of Cx32 and downregulation of Cx43 expression. Our work implicates that Cxs play crucial roles in facilitating differentiation of hepatic progenitors. To the best of our knowledge, this study provides the first evidence of p38 MAPK pathway and Cxs involved in the lineage restriction of rat hepatic progenitors to hepatocytes.

## Methods

### Plasmids, lentivirals, and inhibitors

In order to knockdown rat Cx43, oligonucleotides were synthesized and cloned into the lentiviral vector pSicoR PGK-puro (Addgene, Cambridge, MA, USA), which contains a selection cassette for puromycin. A shRNA against luciferase was subcloned into the same vector as a control (ShCX43 sense:

5’-TAGTTGCTGCTGGACATGAATTCAAGAGATTCATGTCCAGCAGCAACTTTTTTTC-3’; ShCX43 anti-sense:

5’-TCGAGAAAAAAAGTTGCTGCTGGACATGAATCTCTTGAATTCATGTCCAGCAGCAACTA-3’). For overexpression of Cx32 and Cx43 respectively, the amplified rat Cx32 and Cx43 fragments subcloned into the lentiviral vector pBPLV. An empty pBPLV vector was used as a control.

Vesicular stomatitis virus G protein- (VSV-G) pseudotyped lentiviruses were produced using a four-plasmid transfection system as described previously [43]. Cells were infected overnight with recombinant lentivirus using a multiplicity of infection (MOI) of five in the presence of 5 ng/ml polybrene. For knockdown of Cx43, 3 days after infection, cells were selected by culturing in 2 μg/ml puromycin for 3 days and then used for further experiments. For overexpression of Cx32 or the Cx43, GFP^+^ cells were sorted by FACS before further experiments.

To explore the effects of Cx32 on hepatocyte differentiation, 2-aminoethoxydiphenyl borate (2-APB, Tocris, Minneapolis, MN, USA), which has been shown to directly inhibit [44] Cx32 gap junctions in vitro and effectively block [45] hepatic gap junction communication in vivo, were used to test Cx32 relevance.

### WB cells culture and differentiation

The WB cells were grown in RPMI 1640 medium (Gibco, Waltham, MA, USA) supplemented with 10% fetal calf serum (Hyclone, Chicago, IL, USA), 200 IU/ml penicillin and 50 μg/ml streptomycin. Cells were grown in a 37 °C humidified incubator containing 5% CO_2_. For differentiation to hepatocyte-like cells, WB cells were plated on Matrigel (60%) and collagen IV (40%)-coated plastic cell culture dishes at a density of 7 × 10^4^ cells/cm^2^. The cultures were incubated in Hepatocyte Medium (Sciencell, Carlsbad, CA, USA) supplemented with 25 ng/ml hepatocyte growth factor (HGF), 10 ng/ml oncostatin M (OSM), 1 μM dexamethasone (Dex), 5 ng/mL insulin, and 10 ng/ml epidermal growth factor (EGF) for 7 days.

It has been reported that Matrigel could induce differentiation of WB-F344 cells into biliary cells [46, 47]. For Matrigel-induced cholangiocyte differentiation, 50% Matrigel was spread on a six-well plate and allowed to settle for at least 30 min at 37 °C. Cells were then plated at a density of 2 × 10^5^ cells per well and cultured in basal medium supplemented with 5% fetal calf serum (Hyclone), 50 ng/ml epidermal growth factors (PeproTech, Rocky Hill, NJ, USA), 30 ng/ml insulin-like growth factor II (PeproTech) and 10 mg/ml insulin (R&D Systems, Minneapolis, MN, USA) for 5 days.

### Isolation and culture of rat hepatocytes and hepatic stem cells

Fisher 344 rats purchased from Vital River (Beijing, China) were maintained under a constant 12 h light/dark cycle and fed a standard rodent chow and water. Rats weighing 180–200 g were anesthetized with ketamine and xylazine via intraperitoneal and primary hepatocytes were freshly isolated by the two-step liver perfusion method of Seglen [48]. Eighteen-day-pregnant rats were anesthetized in the same way and the protocol for the hepatic stem cell isolation was as described [49]. Briefly, the fetal liver tissue was enzymatically processed by 300 U/ml type IV collagenase and 0.3 mg/ml deoxyribonuclease at 37 °C with frequent agitation for 20 min. Enriched suspensions were pressed through a 75-gauge mesh and spun at 1200 rpm for 5 min before resuspension. Isolated single cells were selectively cultured on 10-cm well plates at 8 × 10^4^ cells/cm^2^ in Kubota’s Medium. The rats were euthanized after the hepatocytes isolation and the fetus extraction.

### Quantitative real time-polymerase chain reaction (qRT-PCR) analysis

Total RNA was extracted using RNeasy Plus Mini Kit (Qiagen, Hilden, Germany) as per the manufacturer’s instructions. Reverse Transcription was carried out with the SuperScript First-Strand Synthesis System for RT-PCR (Invitrogen, Carlsbad, CA, USA). HotStarTaq Master Mix Kit (Bio-Rad, Hercules, CA, USA) was used for PCR. Real-time PCR was carried out in the Bio-Rad IQ5 amplification system (Bio-Rad), and the results were calculated using the delta–delta CT method. PCR primers are listed in Table 1.

**Table 1:** Primers used for real-time PCR

Rat-Cx43-S	TCTGCCTTTCGCTGTAACACT
Rat-Cx43-AS	GGGCACAGACACGAATATGAT
Rat-CX32-S	CCAGGGCTCAAGGTTATTGA
Rat-CX32-AS	TCTCCATCCACAGTGCAGAG
Rat-Afp-S	ACCTGACAGGGAAGATGGTG
Rat-Afp-AS	GCAGTGGTTGATACCGGAGT
Rat-Alb-S	AATTGGCAACAGACCTCACC
Rat-Alb-AS	GCACTGGCTTATCACAGCAA
Rat-CYP1B1-S	CCCGTGGTGGTGCTGAAT
Rat-CYP1B1-AS	AAAGAGGCGAAGGGAGGC
Rat-CK18-S	TTTGCGAATTCTGTGGACAA
Rat-CK18-AS	ACCTCGTGATGTTGGTGTCA
Rat-CK19-S	CCACACTACGCAGATCCAGA
Rat-CK19-AS	ATGCTGAGCTGAGACTGCAA
Rat-Hnf1a-S	ACCAGTCCCACAGTGTCCTC
Rat-Hnf1a-AS	GCCATCTGGGTGGAGATAAA
Rat-Hnf4a-S	AAATGTGCAGGTGTTGACCA
Rat-Hnf4a-AS	CACGCTCCTCCTGAAGAATC
Rat-Ggt4-S	GTCACCAACTTCAACTCTGC
Rat- Ggt4-AS	CCTTATCACTGTTTACCTCGG
Rat-Hnf6-S	GAAAATAAGCGTCCGTCCAAAG
Rat-Hnf6-AS	CTGGCATTCATGAAGAAGTTGC
Rat-GAPDH-S	TGCCACTCAGAAGACTGTGG
Rat-GAPDH-AS	TTCAGCTCTGGGATGACCTT

*S* sense, *AS* anti-sense

### Western blotting

Proteins were extracted from the cultured cells with RIPA buffer (50 mM Tris-HCl. pH 7.5, 150 mM NaCl, 1% NP-40, 0.5% sodium deoxycholate, 0.1% SDS) containing protease inhibitors (Roche, Basel, Switzerland). Protein concentrations were measured using the Bradford method (Bio-Rad) and then 80 g of total lysate from each sample was used to perform Western blot. Antibodies used included sheep anti-rat albumin (Bethyl Laboratories Montgomery, TX, USA), rabbit anti-rat Cyp1b1 (Santa Cruz Biotechnology, Dallas, TX, USA), rabbit anti-rat Cx32 (Abcam, Cambridge, MA, USA), rabbit anti-Rat Cx43 (Abcam), and rabbit anti-phospho-p38 MAPK/p38 MAPK antibodies (Cell Signaling Technology, Danvers, MA, USA).

### Immunostaining

Cells were fixed with 4% paraformaldehyde for 20 min at room temperature and blocked with 10% goat or donkey serum for 1 h. The primary antibodies were incubated with the cells at 4 °C overnight and secondary antibodies were incubated for 45 min at room temperature. After being washed with PBS 3 times, the cells were examined under confocal laser microscope. Confocal images were collected by an LSM 510 META confocal system (Carl Zeiss, Oberkochen, Germany).

### Cell viability assay

Hepatocytes derived from progenitors with overexpression of Connexin 32, knockdown of Connexin 43 or inhibition of p38 MAPK were digested with trypsin, counted, and then plated in a 96-well plate at a density of 1 × 10^3^ per well. The measurement of viable cell mass was performed with a Cell Counting Kit (Dojin Laboratories, Kumamoto, Japan) to count living cells by WST-8.

### Flow cytometry analysis

Hepatocytes derived from progenitors with or without inhibition of p38 MAPK were digested with trypsin, and then centrifuged at 1000 rpm for 3 min. Cells were washed three times with cool PBS and then stained with Annexin V FITC and PI solution for 15 min at room temperature in a dark environment. After adding Annexin V binding solution, the flow cytometry analysis can be carried out within 1 hour.

### Indocyanine green uptake

After the cells were washed with PBS, indocyanine green (ICG) (Sigma-Aldrich, St Louis, MO, USA) solution was added at a final concentration of 1 mg/ml. The cells were incubated with ICG at 37 °C for 30 min and were rinsed three times in PBS, and then were examined microscopically.

### Urea synthesis

The amounts of urea in the culture media were measured after the cells were incubated with 20 mM ammonium chloride. Urea concentrations were determined by QuantiChrom Urea Assay Kit (BioAssay Systems, Hayward, CA, USA) according to the manufacturer’s instructions.

### CYP induction and metabolism assay

To evaluate CYP450 induction, differentiated cells were cultured with 25 μM rifampicin for 3 days, with media changed every day. CYP3A4 activity was quantified using P450-GloTM CYP3A4 Luciferin-IPA kit (Promega, Madison, WI, USA) per manufacturer’s instruction. Total cell numbers were used to normalize the data.

### Animal experiments

Male Fisher 344 rats weighing about 40–50 g were used in all experiments as animal models. All animal work was approved by the Institutional Animal Care and Use Committee (IACUC) at Beijing Institute of Transfusion Medicine (Reference number: IACUC of AMMS-2011-183). Seventy percent partial hepatectomy (PH) was performed. At 30 min before PH surgery, some rats were injected intraperitoneally with a SB203580 (Cell Signaling Technology), dissolved in 0.9% NaCl at a dose of 20 mg/kg body weight. The inhibitor was injected every 2 days. The rats injected DMSO were used as a control.

### Statistical analysis

Data are shown as means and standard deviations. Two-tailed Student’s *t* test was applied for calculating statistical probability in this study. *P* values less than 0.05 were considered to be statistically significant.

## Results

### Cx32 expression was significantly increased but Cx43 expression was dramatically decreased in hepatocyte differentiation of WB cells

**Fig. 1:**
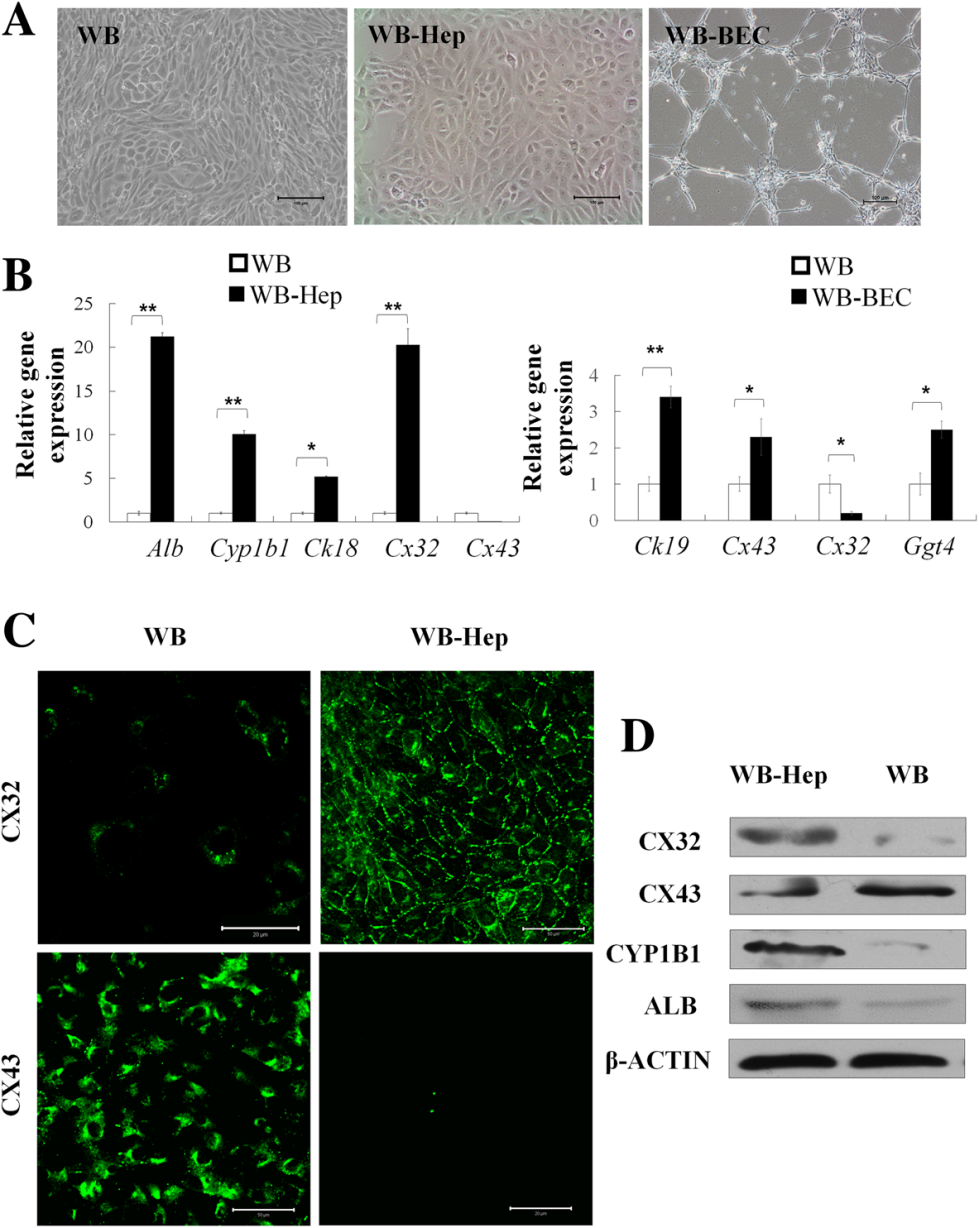
The expression pattern of Cxs in differentiation of WB cells. **a** Representative images of WB cells, hepatocytes (WB-Hep) and biliary epithelial cells (WB-BEC) differentiated from WB cells. **b** qRT-PCR analysis of hepatic and biliary markers in WB-Hep and WB-BEC. **c** Immunostaining of Cx32 and Cx43 in WB cells and WB-Hep. **d** Western blotting of hepatic markers in WB cells and WB-Hep; β-actin was used as an internal control. Scale bars:100 μm for A and 50 μm for C. Data represented as mean ± SEM. ^*^ *p* < 0.05, ^**^ *p* < 0.01

WB cells were small and polygonal and had a high nuclear/cytoplasmic ratio when cultured on plastic in basal medium. After being induced with the hepatic differentiation conditions, the hepatocytes derived from WB cells (WB-Hep) became enlarged and more flattened, and expressed hepatocyte markers *Alb*, *Ck18*, and *Cyp1b1*; but those cells driven toward a biliary epithelial cell fate formed luminal structures, and gained cytoskeratin 19 (*Ck19)* and glutamyl transpeptidase 4 (*Ggt4*) expression, which is specifically induced in biliary cells (Fig. 1a, b). qRT-PCR result showed that WB cells expressed high *Cx43* and barely detectable *Cx32* (Fig. 1b, c), as previously reported [22]. Interestingly, when WB cells were differentiated into hepatocytes, expression of *Cx43* decreased dramatically, while *Cx32* expression was significantly increased. However, when WB cells were induced to biliary epithelial cells, we observed increased *Cx43* expression and decreased *Cx32* expression (Fig. 1b). Furthermore, a shift from Cx43 to Cx32 expression during hepatic differentiation of WB cells was also confirmed by immunofluorescence staining and Western blotting (Fig. 1c, d).

### Cx32 and Cx43 were involved in the hepatic lineage restriction

**Fig. 2:**
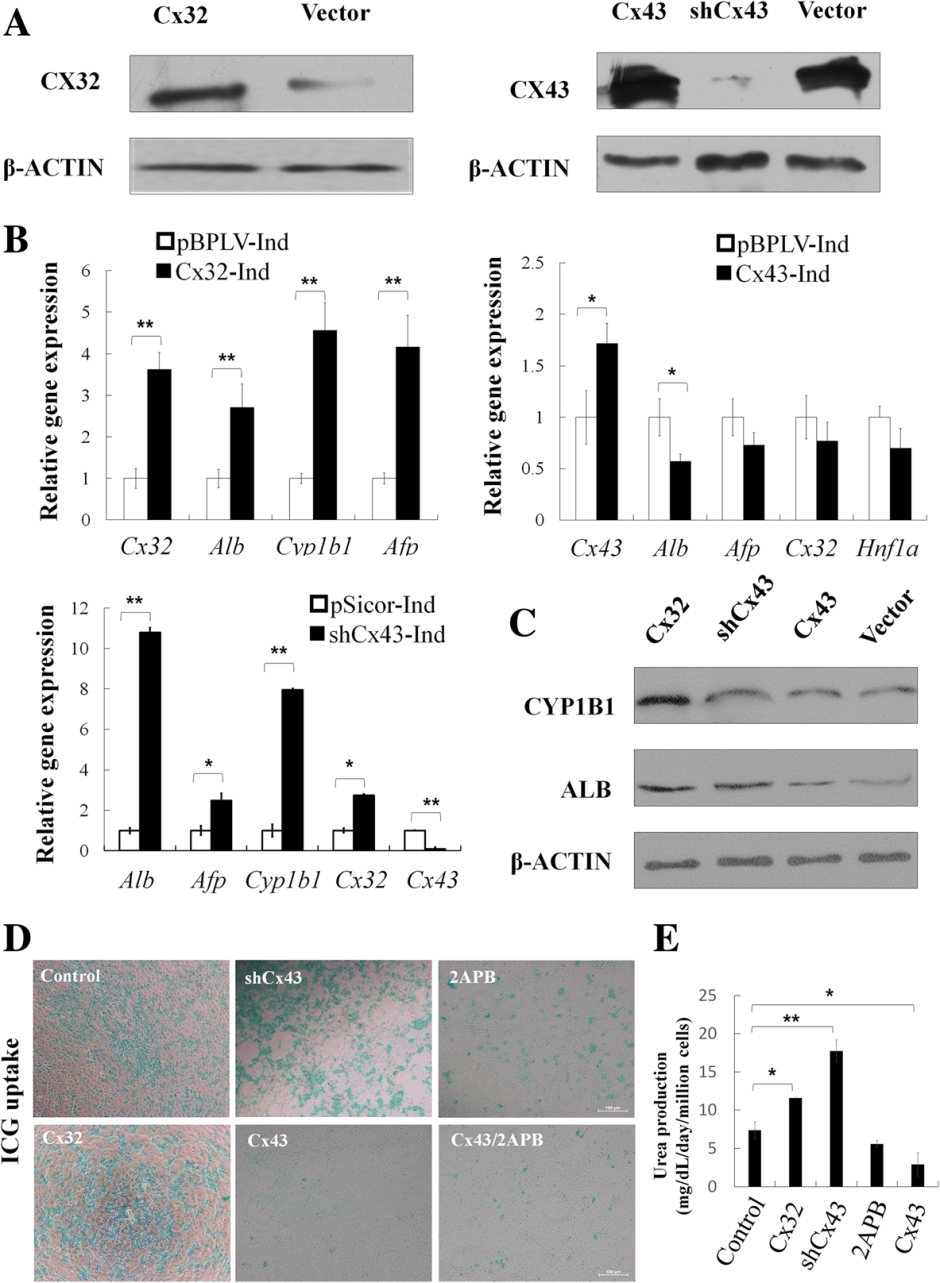
Hepatocyte differentiation from hepatic progenitor cells was affected by Cxs expression. **a** Overexpression of Cx32 and Cx43, and knockdown of Cx43 in WB cells were confirmed by Western blotting. **b** qRT-PCR analysis of hepatic markers in cells differentiated from WB cells with Cx32 or Cx43 overexpression, or Cx43 knockdown. **c** Western blotting of hepatic markers in cells differentiated from WB cells with Cx32 or Cx43 overexpression, or Cx43 knockdown. **d** ICG uptake. **e** Urea production of WB cells with Cx32 or Cx43 overexpression, 2APB, or Cx43 knockdown. Scale bars: 100 μm. Data represented as mean ± SEM. ^*^ *p* < 0.05, ^**^ *p* < 0.01

To investigate the roles of Cx32 and Cx43 during the differentiation of hepatic progenitor cells*,* in vitro gain and loss of function analyses were performed in WB cells. Recombinant lentivirus expressing Cx32, Cx43, and Cx43-siRNA were used to achieve efficient overexpression of Cx32 and Cx43, and knockdown of Cx43 in WB cells (Fig. 2a). Under conditions for hepatocyte differentiation, cells with Cx32 overexpression showed much higher expression levels of hepatocyte markers [Alb, alpha fetoprotein (Afp), and Cyp1b1] than vector control. In contrast, overexpression of Cx43 decreased hepatocyte-specific genes. These results demonstrated that Cx32 overexpression promotes hepatocyte differentiation, whereas Cx43 overexpression inhibits hepatocyte differentiation. In addition, Cx43-supressing siRNA transfection upregulated the expression levels of hepatocyte markers, suggesting that Cx43 knockdown promotes hepatocyte differentiation (Fig. 2b, c). On the other hand, under conditions for cholangiocyte differentiation, cells with Cx43 overexpression combined with 2APB treatment showed much higher expression levels of biliary markers *Ggt4*, *Ck19* and *Hnf6* than vector control (Additional file 1: Figure S1A).

For function analysis, ICG uptake was tested. Most of cells turned to deep green after incubation with ICG for 30 min in Cx32 overexpression or Cx43 knockdown group, whereas few green cells appeared in Cx43 overexpression, 2APB, Cx43 overexpression plus 2APB group (Fig. 2d). In addition, after incubation with 20 mM ammonium chloride, urea production in cell culture supernatant was much higher in Cx32 overexpression or Cx43 knockdown groups than that in the control group, and was lower in the 2-APB or Cx43 overexpression group (Fig. 2e). Taken together, these findings show that Cx32 and Cx43 are closely correlated with the differentiation of bi-potential hepatic progenitor cells.

### Inhibition of p38 MAPK activity promoted the differentiation of hepatic progenitor cells associating with modulation of Cx32 and Cx43

**Fig. 3:**
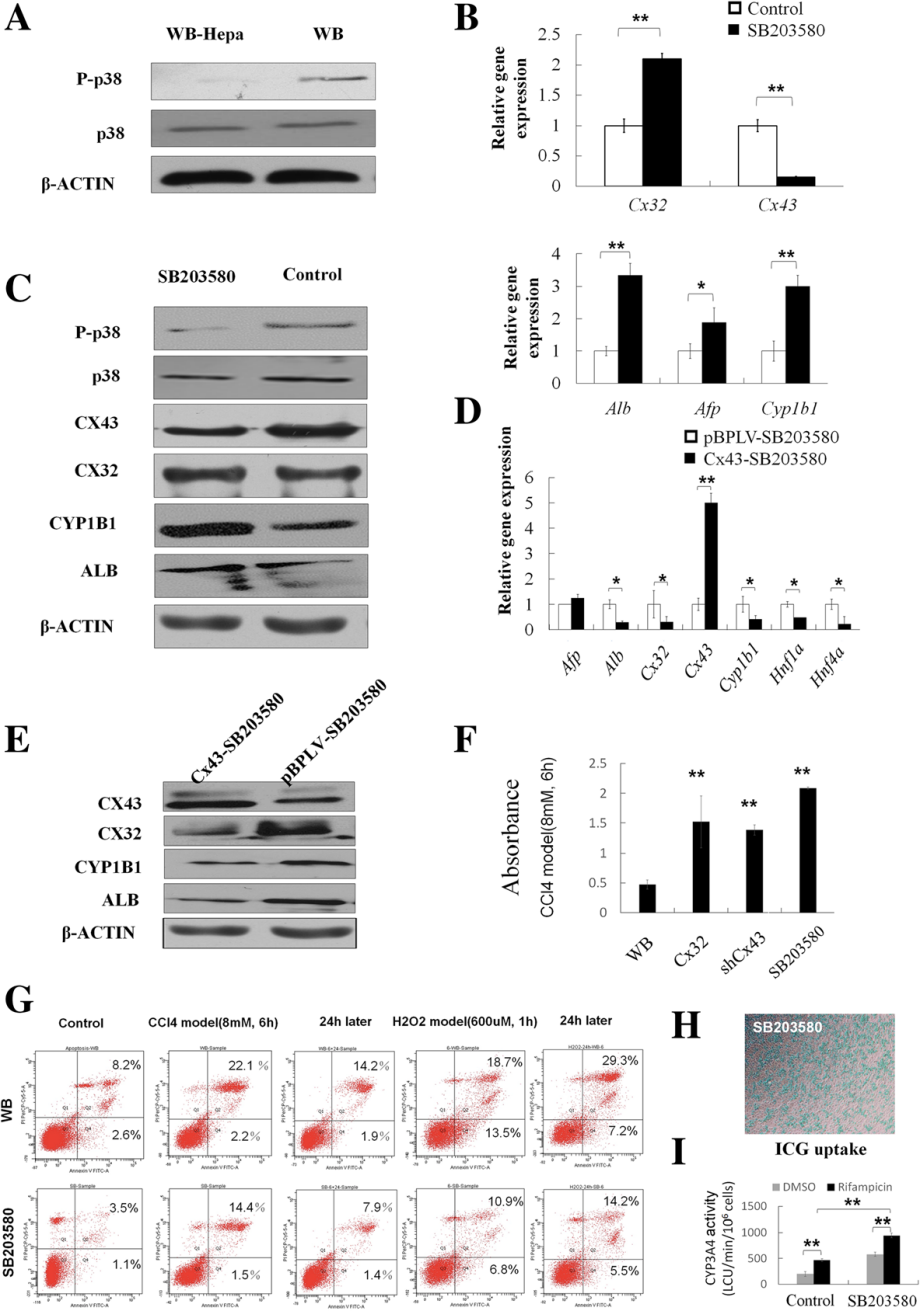
p38 MAPK pathway affected hepatocytes differentiation associating with downregulation of Cx43 and upregulation of Cx32. **a** Western blotting of phospho-p38 (P-p38) and total p38 in WB cells and WB-Hep. **b** qRT-PCR analysis of WB-Hep induced with or without SB203580 treatment. **c** Western blotting of WB-Hep induced with or without SB203580 treatment. **d** qRT-PCR analysis of WB-Hep induced with SB203580 from WB cells and Cx43-overexpressed WB cells. **e** Western blotting of WB-Hep induced with SB203580 from WB cells and Cx43-overexpressed WB cells. **f** Cell viability test by CCK8. **g** Flow cytometry analysis of apoptosis. **h** ICG uptake. **i** Induction of CYP3A4 activity in response to 72 hours of stimulation with PXR agonist rifampicin. Data represented as mean ± SEM. ^*^ *p* < 0.05, ^**^ *p* < 0.01

During the process of hepatic progenitors differentiating into hepatocytes, phosphorylation level of p38 MAPK was decreased, compared to total levels of p38 MAPK (Fig. 3a), which suggested that p38 MAPK activity was negatively related to hepatocyte differentiation. Moreover, when a p38 MAPK inhibitor SB203580 was introduced in the hepatocyte differentiation medium, hepatocytes derived from WB cells expressed higher levels of hepatocyte markers Alb and Cyp1b1, with downregulation of Cx43 and upregulation of Cx32, as shown by qRT-PCR and Western blotting assays (Fig. 3b, c). Whereas, the expression of hepatocyte markers was much lower in WB cells with Cx43 overexpression, even under the treatment of SB203580, indicating that the enhancement of hepatocyte differentiation by SB203580 was compromised by Cx43 overexpression (Fig. 3d, e).

In addition, to further confirm the closed relationship between p38 MAPK pathway and modulation of Cxs in vivo, rats were injected intraperitoneally with SB203580 at 30 min before PH surgery. After that, the expression of Cx43 was downregulated, whereas that of Cx32 was upregulated (Additional file 1: Figure S1B).

To further assess the effectiveness of SB203580 under stressful stimuli, CCL4-induced hepatocytes damage model and H2O2 induced oxidative damage model in vitro were established. Incubation of cells with 8 mM CCL4 or 600uM H2O2 for 1 h resulted in apoptosis and necrosis in hepatocytes derived from WB cells (24.3% and 32.2% respectively) but obvious decrease under the treatment of SB203580 as shown by the results of a MTT assay and flow cytometry, indicating that SB203580 can resist the cytotoxicity, reduce the apoptosis, promote proliferation and then protect against hepatocyte injury (Fig. 3f and g). For function analysis, ICG uptake was tested. Most of cells turned to deep green after incubation with ICG for 30 min in SB203580 group (Fig. 3h). Importantly, a sensitive and selective bioluminescent assay also confirmed CYP3A4 activity, which was inducible by rifampicin and improved by SB203580 (Fig. 3i).

### Cx32/Cx43 regulated hepatocytes differentiation via Hnf1a and Hnf4a

**Fig. 4:**
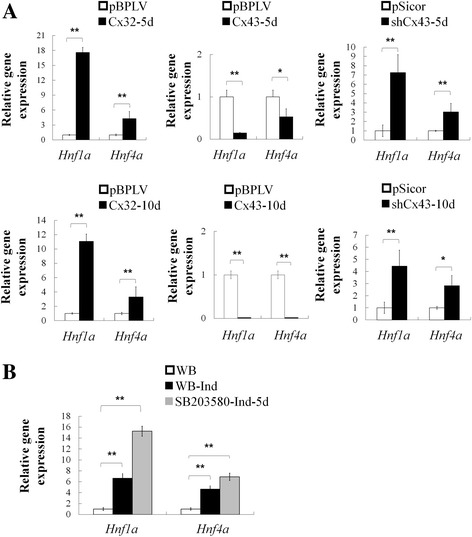
p38 MAPK pathway and expression level of Cxs have an effect on Hnf1a and Hnf4a in differentiation of hepatic progenitors. **a** qRT-PCR analysis of Hnf1a and Hnf4a expression in WB-Hep at day 5 and day 10 induced from WB cells with Cx32 or Cx43 overexpression, or Cx43 knockdown. **b** qRT-PCR analysis of Hnf1a and Hnf4a expression in WB cells and WB-Hep induced with or without SB203580 treatment. Data represented as mean ± SEM. ^*^ *p* < 0.05, ^**^ *p* < 0.01

Hepatocyte nuclear factors (HNFs) are a group of important transcription factors that regulate liver-specific gene expression. We found that expression of *Hnf1a* and *Hnf4a* were significantly upregulated in hepatocytes differentiated from WB cells with Cx32 overexpression or Cx43 knockdown, while downregulated in cells with Cx43 overexpression (Fig. 4a). Furthermore, *Hnf1a* and *Hnf4a* expression were greatly enhanced during hepatocyte differentiation of WB cells with the treatment of SB203580 (Fig. 4b). These data offer circumstantial evidence for critical roles of Cxs and p38MAPK pathway in hepatocyte differentiation.

### p38 MAPK affected differentiation of fetal hepatic progenitor cells

**Fig. 5:**
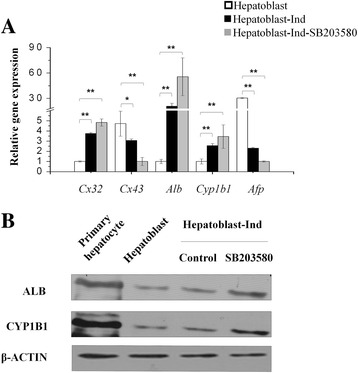
p38 MAPK pathway affects differentiation of fetal rat hepatic progenitor cells. **a** qRT-PCR analysis of fetal rat hepatic progenitor cells and their derivation with or without SB203580 treatment. **b** Western blotting analysis of rat hepatic progenitor cells and their derivation with or without SB203580 treatment. Data represented as mean ± SEM. ^*^ *p* < 0.05, ^**^ *p* < 0.01

To verify the intimate relationship between p38 MAPK pathway and Cxs expression ex vivo, primary hepatic progenitor cells were isolated from fetal rat livers, while primary rat hepatocytes were isolated from adult liver as control (Additional file 1: Figure S1C). When fetal rat hepatic progenitor cells were differentiated into hepatocytes, Cx32 expression was upregulated, while Cx43 expression was downregulated. After treatment by SB203580, primary hepatic progenitor cells expressed higher levels of hepatocyte markers Alb and Cyp1b1 associating with down-regulation of Cx43 and upregulation of Cx32 (Fig. 5a, b).

## Discussion

Stem cells represent a promisingly potential source of cells for regeneration and it is quite important to understand the mechanism involved in stem cell differentiation. Until now, it has not been reported the essential relationship and mechanism between Cxs and differentiation of rat hepatic progenitors. In the present study, we confirm critical roles of Cxs on differentiation. Furthermore, we reveal that p38 MAPK pathway play a significant effect on lineage restriction of rat hepatic progenitors to hepatocytes associating with modulation of Cx32 and Cx43.

GJIC maintains normal growth and differentiation of cells in many tissues. In rat liver, Cx32, a fundamental element of GJIC, becomes detectable in the late stages of gestation and reaches maximal levels about 1 week after birth [50]. In addition, most of liver-specific functions are known to be related with Cx32 expression such as albumin secretion, ammonia detoxification, glycogenolysis, bile secretion, and xenobiotic phase I biotransformation. Most importantly, Cx expression patterns undergo lineage stage-dependent transformation in embryonic liver. The above phenomena hint that Cxs might play important roles in development and maturation of hepatic progenitor cells. In our study, qRT-PCR and Western blotting assays showed that WB cells expressed high level of Cx43 and barely detectable Cx32. When they differentiated into hepatocytes under the induction condition, Cx32 expression was dramatically increased while Cx43 expression was disappeared, which drew out a correlation between cell differentiation and the transformation of Cx expression patterns. To determine roles of Cx32/Cx43 in regulating differentiation of hepatic progenitor cells, we established stable WB cell lines expressing Cx32/Cx43 and shRNAs targeted-Cx43. In contrast to control cells, Cx32-overexpression and Cx43-downregulation cells more efficiently differentiated into cells that expressed Alb, Cyp1b1 protein. Phenotypic and functional analyses provided more convincing evidence that Cxs are critical mediators in differentiation of hepatic progenitor cells.

The p38 MAPK pathway widely involved in development, regulating a large number of processes, including growth, embryonic development, and tissue homoeostasis [51]. Previous studies showed p38 MAPK activity in liver cancer tissue is significantly higher than that in the adjacent tissue and activation of p38MAPK signaling has an association with the invasive or metastatic potential in human hepatocellular carcinoma cells, which suggested that inactivation of p38 occurs in the normal/mature hepatocytes [52]. Besides, Cx32 was investigated in connection with p38 in rat hepatocytes [34]. Yang et al. also found p38 MAPK signaling pathway may be functionally related to regulation of gap junction in rat neuronal stem cell-derived cells [36]. More interestingly, when p38 MAPK pathway was activated during partial hepatectomy associated with downregulation of Cx32, which can be reversed by SB203580 treatment [37]. More recently, some studies showed Cx32 protein decreased in primary cultures of rat hepatocytes following the treatment with a p38 MAPK activator, anisomycin [53]. Our findings showed inhibition of p38 MAPK pathway can promote hepatocyte differentiation from hepatic progenitor cells associating with upregulation of Cx32 expression and downregulation of Cx43 expression. To further confirm the closed relationship between p38 MAPK pathway and modulation of Cxs in vivo, we examined Cx expression in rat livers after 70% partial hepatectomy (PH) by using a p38 MAPK inhibitor SB203580. With SB203580 treatment, upregulation of Cx32 expression and downregulation of Cx43 expression were observed at 24 hours after PH. Apart from verification in vitro and in vivo, we also demonstrated our hypothesis by ex vivo analysis. Our results showed that inhibition of p38 MAPK pathway remarkably improve hepatocytes differentiation from freshly isolated rat fetal hepatic progenitor cells accompanied by a shift from Cx43 to Cx32 expression.

## Conclusions

In summary, we demonstrate that Cxs play crucial roles in facilitating differentiation of hepatic progenitors. Also, p38 MAPK pathway has a strong bond with Cxs modulation, which highly affects the last phase of hepatic lineage restriction. Our work further implicates that regulators of Cxs and their related pathways might provide new insights to improve lineage restriction of stem cells to mature hepatocytes. With further efforts, we expect to use this clue to derive functional hepatocytes for widely medical applications. Certainly, it will also be a new starting point for understanding of hepatic differentiation.

## Additional file

Additional file 1: Figure S1. (A) qRT-PCR analysis of biliary markers in cells differentiated from WB cells with Cx43 overexpression combined with 2APB treatment. (B) Western blotting for phospho-p38 (P-p38), total p38, Cx32, and Cx43 in rat livers treated with or without SB203580 for 24 h after partial hepatectomy. Each condition was spliced from a single gel to remove intervening lanes. (C) Morphology of primary hepatocytes derived from adult rat livers and hepatoblasts isolated from fetal rat livers. Scale bars:100 μm, 50 μm, 25 μm for B. Data represented as mean ± SEM. ^*^ *p* < 0.05, ^**^ *p* < 0.01. (TIF 4003 kb)

## Abbreviations

Afp: Alpha fetoprotein
Alb: Albumin
Ck: Cytoskeratin
Cx: Connexin
cytochrome P450: CYP
Dex: Dexamethasone
EGF: Epidermal growth factor
ESCs: Embryonic stem cells
Ggt4: Glutamyl transpeptidase 4
GJIC: Gap junctional intercellular communication
HGF: Hepatocyte growth factor
HNFs: Hepatocyte nuclear factors
IACUC: Institutional Animal Care and Use Committee
ICG: Indocyanine green
iPSCs: Induced pluripotent stem cells
MOI: Multiplicity of infection
OSM: Oncostatin M
p38 MAPK: p38 mitogen-activated protein kinase
PH: Partial hepatectomy
PSCs: Pluripotent stem cells
VSV-G: Vesicular stomatitis virus G protein
WB cells: WB-F344 cells
WB-BEC: Biliary epithelial cells
WB-Hep: Hepatocytes derived from WB cells
