# Supplementary figures and images for: Connexin 32 and connexin 43 are involved in lineage restriction of hepatic progenitor cells to hepatocytes
Source: Stem Cell Res Ther. 2017 Nov 7;8:252. doi: 10.1186/s13287-017-0703-2 (PMC5678556; doi:10.1186/s13287-017-0703-2)

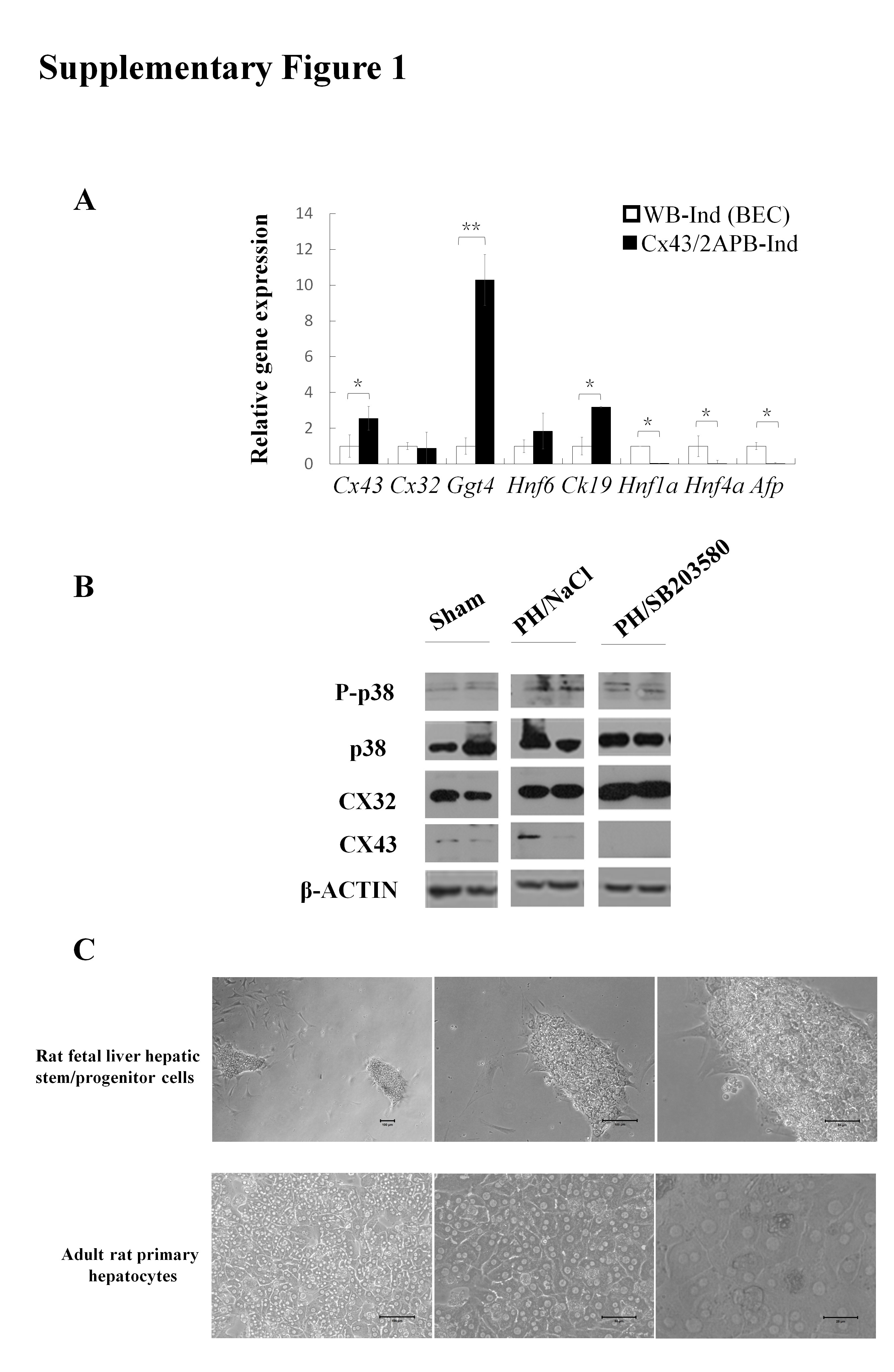

Supplement: Additional file 1: Figure S1. — (A) qRT-PCR analysis of biliary markers in cells differentiated from WB cells with Cx43 overexpression combined with 2APB treatment. (B) Western blotting for phospho-p38 (P-p38), total p38, Cx32, and Cx43 in rat livers treated with or without SB203580 for 24 h after partial hepatectomy. Each condition was spliced from a single gel to remove intervening lanes. (C) Morphology of primary hepatocytes derived from adult rat livers and hepatoblasts isolated from fetal rat livers. Scale bars:100 μm, 50 μm, 25 μm for B. Data represented as mean ± SEM. * p < 0.05, ** p < 0.01. (TIF 4003 kb) [file 13287_2017_703_MOESM1_ESM.tif]
